# Therapies for Membranous Nephropathy: A Tale From the Old and New Millennia

**DOI:** 10.3389/fimmu.2022.789713

**Published:** 2022-03-01

**Authors:** Francesco Scolari, Federico Alberici, Federica Mescia, Elisa Delbarba, Hernando Trujillo, Manuel Praga, Claudio Ponticelli

**Affiliations:** ^1^ Department of Medical and Surgical Specialties, Radiological Sciences and Public Health, University of Brescia, Brescia, Italy; ^2^ Nephrology Unit, Spedali Civili Hospital, ASST Spedali Civili di Brescia, Brescia, Italy; ^3^ Department of Nephrology, Hospital Universitario 12 de Octubre, Madrid, Spain; ^4^ Department of Nephrology, Instituto de Investigación Hospital Universitario 12 de Octubre, Madrid, Spain; ^5^ Independent Researcher, Milano, Italy

**Keywords:** membranous nephropathy, glomerulonephitis, nephrotic syndome, rituximab, cyclical therapy

## Abstract

Primary Membranous Nephropathy (PMN) is the most frequent cause of nephrotic syndrome in adults. If untreated, PMN can lead to end-stage renal disease; moreover, affected patients are at increased risk of complications typical of nephrotic syndrome such as fluid overload, deep vein thrombosis and infection. The association of PMN with *HLA-DQA1* and the identification in around 70% of cases of circulating autoantibodies, mainly directed towards the phospholipase A2 receptor, supports the autoimmune nature of the disease. In patients not achieving spontaneous remission or in the ones with deteriorating kidney function and severe nephrotic syndrome, immunosuppression is required to increase the chances of achieving remission. The aim of this review is to discuss the evidence base for the different immunosuppressive regimens used for PMN in studies published so far; the manuscript also includes a section where the authors propose, based upon current evidence, their recommendations regarding immunosuppression in the disease, while highlighting the still significant knowledge gaps and uncertainties.

## Introduction

Membranous nephropathy (MN) is a disease induced by deposition of immune complexes in the subepithelial space of the glomerulus (1). In the primary form (PMN), immune complex formation is driven by autoantibodies, with the most frequent autoantigen being the M type phospholipase A2 receptor (PLA2R) (2), a protein normally expressed in podocytes. Of note, other antigens may be involved as well (3–6). The genetic association of PMN with *HLA-DQA1 (* 7) supports abnormal antigen presentation as a further key step contributing to disease. Yet, several aspects in the pathogenesis remain unclear, such as what are the mechanisms that induce loss of self-tolerance, how exactly the IgG4 autoantibodies cause podocyte injury and proteinuria, how the complement system is activated, and whether or not it plays a role in podocyte damage (8).

From a clinical point of view, the natural course of PMN is variable. Several retrospective studies with relatively short follow-up, including patients with and without nephrotic range proteinuria, reported that about 1/3 of patients do not present nephrotic syndrome or may enter a spontaneous remission, 1/3 maintain stable kidney function, with proteinuria fluctuating between nephrotic and sub-nephrotic range, while the remaining tend to have persistent nephrotic syndrome and progress to end-stage renal disease (ESRD) (9–14).

Studies with long-term follow-up (10 years or more) clearly showed that kidney survival is significantly affected by the length of patients’ observation. In an Italian randomized controlled trial in patients with biopsy-proven PMN and nephrotic syndrome at presentation, after 10 years of follow-up only 5% of the cases assigned to symptomatic treatment were in complete remission, and another 28% were in partial remission (for more details on the definition of complete and partial remission, please see the **Appendix**). Of note, in the same group, 40% of patients developed ESRD or died within 10 years from randomization (15). In a systematic review including all studies published up to 1994, Hogan et al. (16) found that the kidney survival of patients with MN averaged around 50% at 14 years. DuBuf-Vereijken et al. (17) analysed the reports published during the previous 25 years, excluding patients with a follow-up shorter than 3 years, and identified a 100% renal survival rate in non-nephrotic patients; on the other hand, nearly 50% of patients with PMN and nephrotic syndrome experienced deterioration in kidney function. An increased risk of chronic kidney disease (CKD) progression is only one of the risks of persistent nephrotic syndrome, which is also associated with a number of severe extra-renal complications including vascular thrombosis, infection, and cardiovascular disease. Non-immunomodulatory and non-specific nephroprotective approaches, although necessary, are therefore not sufficient for patients with PMN and persistent or severe nephrotic syndrome. In this context, a role for immunomodulation has been proposed, in order to try to modify disease progression. The aim of this manuscript is to review evidence of historical and emerging immunomodulatory approaches for the treatment of PMN, with a particular focus on prospective randomised controlled studies (**Table 1**).

**Table 1:** Randomized controlled studies in Primary Membranous Nephropathy.

Trial (year)	Interventions, number of patients	Follow-up (months)	CR+PR (as per study definition)	p	Relapse rate	p	Adverse events (number)	p	
**Corticosteroids or alkylating agents versus supportive treatment**									
**Donadio JV et al. (1974)** (18)	Oral CYC (1.5 – 2.5 mg/kg/day) for 1 year (n= 11) Placebo (n=11)	12 from baseline	4/11^$^ 2/11^$^	NA	NA	NA	Leukopenia (n=5); nausea (n=4); partial alopecia (n=3)	NA	
**Collaborative Study of the Adult Idiopathic Nephrotic Syndrome (1979)** (19)	PDN 100-150 mg/every other day (according to body weight) tapered in case of response otherwise withdrawn (n=34) Supportive treatment (n=38)	Mean: 23 (range, 4-52)	22/34^%^ 11/38^%^	0.002	8/22 NA	NA	Gastrointestinal bleeding (n=1) Psychiatric alterations (n=1)	ns	
**Cattran DC et al. (1989)** (20)	PDN on alternate days (45 mg per square meter of BSA) for 6 months (n=81) Supportive treatment (n=77)	Mean: 48 ± 3.2	Similar proportion throughout the follow-up	ns	NA	NA	Cushingoid features (n=12); glucose intolerance (n=4); mood swings (n=5); excessive weight gain (n=3); gastrointestinal disturbances (n=2); acne (n=2); muscle weakness (n=2); headache (n=1); excessive hair loss (n=1); death (myocardial infarct, n=1) NA	NA	
**Cameron JS et al. (1990)** (21)	PDLN 125 – 150 mg every other day (according to body weight) for 8 weeks (n=52) Placebo (n=51)	Mean: 52	10/52* 7/51*	ns	NA	NA	pulmonary embolism (n=2); stroke (n=2); death (n=1); duodenal ulcer (n=1); peripheral neuropathy (n=1); intracranial hypertension (n=1) neoplasia (n=2, 1 death); perforated ischemic bowel (n=1, 1 death) death for uncertain causes (n=1)	NA	
**Cyclical regimen versus supportive treatment**									
**Ponticelli C et al. (1984)** (22)	IV MPDN for three days followed by oral MPDN (0.4 mg/kg/day) or PDN (0.5 mg/kg/day) for 27 days alternate to chlorambucil (0.2 mg/kg/day) for one month. Overall duration 6 months (n=33) Supportive treatment (n=30)	Mean: 31.4 ± 18.2 Mean: 31.4 ± 18.2	23/32* 9/30*	0.001	3/26^£^ NA	NA	Peptic ulcer (n=1); gastric intolerance (n=1); glucose intolerance (n=1) 2 drop out: progressive renal failure (n=1); high titer of anti-dsDNA (n=1)	NA	
**Ponticelli C et al. (1989)** (23)	IV MPDN (1g/day) for three days followed by oral MPDN (0.4 mg/kg/day) or PDN (0.5 mg/kg/day) for 27 days alternate to chlorambucil (0.2 mg/kg/day) for one month. Overall duration 6 months (n=42) Supportive treatment (n=39)	Median: 60 (range, 24–132)	28/42* 9/39*	NA	NA	NA	Gastrointestinal disturbances (n=3); peptic ulcer (n=2); tremors (n=2); leukopenia n=2); cramps (n=2); infections (n=1); anxiety (n=1); liver disfunction (n=1); diabetes mellitus (n=1); obesity (n=1)	NA	
**Ponticelli C et al. (1995)** (15)	IV MPDN (1g/day) for three days followed by oral PDN (0.5 mg/kg/day) for 27 days alternate to chlorambucil (0.2 mg/kg/day) for one month. Overall duration 6 months. The cycle may be repeated after at least two years from the first treatment (n=42) Supportive treatment (n=39)	Up to 10 years	26/42* 13/39*	NA	4/35^%^ NA	NA	Peptic ulcer (n=2); leukopenia (n=2); tremors (n=2); cramps (n=2); infections (n=1); gastric intolerance (n=1); anxiety (n=1); liver disfunction (n=1); obesity (n=1); diabetes mellitus (n=1); death (neoplasia, n=1) Death (cardiac arrest hepato-renal failure, cardiac infarct, n=3)	NA	
**Jha V et al. (2007)** (24)	IV MPDN (1g/day) for three days followed by oral PDLN (0.5 mg/kg/day) for 27 days alternate to oral CYC (2 mg/kg/day) for one month. Overall duration 6 months. (n= 47) Supportive treatment (n=46)	Median: 132 (range, 126 – 144)	34/47^%^ 16/46^%^	<0.0001	8/34^%^ 4/16^%^	NA	Infections (n=10) leukopenia (n=3); thrombotic events (n=3); death cardiac event, n=1) Infections (n=14); thrombotic events (n=4); death (advanced uraemia n=2, and pneumonia n=1)	ns	
**Cyclical regimen versus active treatment**									
**Ponticelli C et al. (1992)** (25)	IV MPDN (1g/day) for three days followed by oral MPDN (0.4 mg/kg/day) for 27 days alternate to chlorambucil (0.2 mg/kg/day) for one month. Overall duration 6 months (n=45) IV MPDN (1g/day) for 3 days at the beginning of month 1, 3 and 5 followed by oral MPDN (0.4 mg/kg every other day). Overall duration of 6 months (n=47)	Mean: 54 ± 16 Mean: 54 ± 17	28/45* 18/47*	<0.05 for the first 3 years of follow-up	NA	NA	Gastric discomfort (n=6); leukopenia (n=2); infections (n=2); amenorrhea (n=2) fever (n=1); liver disfunction (n=1); acne (n=1); mild myoclonus (n=1); diabetes mellitus (n=1); death (neoplasia, n=1) Gastric discomfort (n=5); acne (n=2); thrombotic event (n=1); cushingoid appearance (n=1); diabetes mellitus (n=1); death (neoplasia, n=1)	NA
**Ponticelli C et al. (1998)** (26)	IV MPDN (1g/day) for three days followed by oral MPDN (0.4 mg/kg/day) for 27 days alternate to chlorambucil (0.2 mg/kg/day) for one month. Overall duration 6 months. (n= 50) IV MPDN (1g/day) for three days followed by oral MPDN (0.4 mg/kg/day) for 27 days alternate to oral CYC (2.5 mg/kg/day) for one month. Overall duration 6 months. (n= 45)	Median: 36 (range, 12–78) Median: 42 (range 12 – 72)	36/44^%^ 40/43^%^	0.116	11/36^%^ 10/40^%^	NA	Infections (n=6); leukopenia (n=2); bone marrow hypoplasia (n=1); nausea (n=1); glucose intolerance (n=1); amenorrhea (n=1); neoplasia (n=1) Nausea (n=1); cerebral transient ischemic attack (n=1); glucose intolerance (n=1); neoplasia (n=1)	NA	
**Branten AJ et al. (1998)** (27)	IV MPDN (1g/day) for three days followed by oral PDN (0.5 mg/kg/day) for 27 days alternate to chlorambucil (0.15 mg/kg/day) for one month. Overall duration 6 months. (n= 15) Corticosteroids at a comparable dose plus oral CYC (1.5–2 mg/kg/day) for 1 year (n= 17)	Median: 38 (range, 8 – 71) Median: 26 (range, 5 – 68)	2/15* 11/17*	<0.01	NA	NA	Infections (n=8); leukopenia (n=8); thrombocytopenia (n=3); anaemia (n=2); osteonecrosis (n=1); renal artery stenosis (n=1) Infections (n=5); leukopenia (n=3); anaemia (n=2); nausea (n=2); general malaise (n=1)	<0.01	
**Ponticelli C et al. (2006)** (28)	IV MPDN (1g/day) for three days followed by oral MPDN (0.4 mg/kg/day) for 27 days alternate to oral cytotoxic agents (CYC 2.5 mg/kg/day or chlorambucil 0.2 mg/kg/day) for one month. Overall duration 6 months (n= 16) Synthetic ACTH (max 1mg twice per week) for 12 months (n= 16)	Mean: 21.8 ± 7.5 Mean: 21.8 ± 7.6	12/16* 14/16*	ns	7/15^%^ 3/14^%^	ns	Leukopenia (n=2); glucose intolerance (n=2) Hyperpigmentation of the skin (n=6); glucose intolerance (n=2); dizziness (n=1); diarrhea (n=1); onycodystrophy (n=1); folliculitis (n=1)	NA	
**Howman A et al. (2013)** (29)	IV MPDN (1g/day) for three days followed by oral PDLN (0.5 mg/kg/day) for 28 days alternate to chlorambucil (0.15 mg/kg/day) for one month. Overall duration 6 months. (n= 33) 12-month course of CyA (5 mg/kg, target through levels 100–200 μg/L.) (n=36) Supportive treatment (n=37)	Until the achievement of the primary endpoint (maximum of 3 years)	Lowest decline of renal function and highest decline of proteinuria in the cyclic regimen arm	0·003	NA	NA	20/33 pts^%^: haematological events (n=28); metabolic events (n=8); dermatological events (n=4); cardiovascular events (n=4); neurological events (n=3); infections (n=3); gastroenterological events (n=3); renal events (n=1) 18/37 pts^%^: infections (n=8); neurological events (n=6); haematological events (n=5); renal events (n=5); gastroenterological events (n=3); cardiovascular events (n=3); dermatological events (n=2); metabolic events (n=1) 16/38 pts^%^: metabolic events (n=5); neurological events (n=4); haematological events (n=3); cardiovascular events (n=3); renal events (n=2); gastroenterological events (n=2) infections (n=2)	NA	
**Ramachandran R et al. (2017)** (30)	TAC (0.1 mg/kg/day, target trough levels 5–10 ng/ml in the first 6 months and 4–8 ng/mL in the next 6 months) for 12 months and oral PDLN (0.5 mg/kg/day) for 6 months, then tapered (n=35) IV MPDN (1 g/day) for 3 days followed by oral PDLN (0.5 mg/kg/day) for 27 days alternate to oral CYC (2 mg/kg/day) for one month. Overall duration: 6 months (n=35)	24 from baseline	21/35* 30/35*	0.03	10/23* 2/31*	0.0069	Infections (n=16); nephrotoxicity (n=8)^#^; diabetes mellitus (n=6); gastrointestinal disturbances (n=5); tremor (n=4); hypertension (n=3) Infections (n=13); amenorrhea (n=5) ^#^; diabetes mellitus (n=5); leukopenia (n=4); gastrointestinal disturbances (n=3); hypertension (n=2)	^#^<0.05 Others: ns	
**CNIs versus supportive treatment**									
**Cattran DC et al. (1995)** (31)	CyA (3.5 mg/kg/day, target through levels 110–170 μg/L) (n=9) Placebo (n=8)	Mean: 49 (range, 17–75) Mean: 48 (range, 25–88)	6/8* 0/8*	NA	NA	NA	Hypertension (n=6); gastrointestinal disturbances (n=2); infections (n=1); tremor (n=1); hirsutism (n=1) Infections (n=3); hypertension (n=3) gastrointestinal disturbances (n=1); tremor (n=1); hirsutism (n=1)	ns	
**Praga M et al. (2007)** (32)	TAC (0.05 mg/kg/day, target through level 3 – 5 ng/ml, to be increased to 5 – 8 ng/ml in case of lack of response at 2 months; full dose for 12 months, followed by tapering in 6 months) Overall duration: 18 months (n= 25) Supportive treatment (n= 23)	Up to 30 from baseline	19/25^$^ 6/23^$^	0.003	9/19* NA	NA	Glucose intolerance (n=4); diarrhea (n=2); nausea (n=1); headache (n=1); tremor (n=1); gouty arthritis (n=1) Glucose intolerance (n=2); chest pain (n=2); infections (n=1)	ns	
**CNIs versus active treatments**									
**Cattran DC et al. (2001)** (33)	Cya (3.5 mg/kg/day, target through level 125–225 μg/L) for 7 months and low-dose PDN (0.15 mg/kg/day, max 15 mg/day for 26 weeks, withdrawn at week 32) (n=28) Placebo and low-dose PDN (0.15 mg/kg/day, max 15 mg/day for 26 weeks, withdrawn at week 32) (n=23)	Up to 78	11/28* 3/23*	0.007	10/21^%^ 3/5^%^	NA	Hypertension (n=10); nausea (n=4) Hypertension (n=5); nausea (n=1)	NA	
**Chen M et al. (2010)** (34)	TAC (0.1 mg/kg/day, target through level 5–10 ng/ml for the first 6 months, then 2–5 ng/ml for 3 months) and PDN (1mg/kg/day for 1 month, withdrawn at month 8). (n=39) Oral CYC (100 mg/day for 4 months) and PDN (1mg/kg/day for 1 month, withdrawn at month 8). (n= 34)	12 from baseline	31/39* 23/34*	ns	6/33^%^ 5/23^%^	ns	Glucose intolerance/diabetes (n=12) ^#^; infections (n=8) ^#^, 3 SAE; liver disfunction (n=7, 1 SAE); hypertension (n=5) ^#^; gastrointestinal symptoms (n=3, 2 SAE); tremor (n=3); transient worsening of renal function (n=1) Liver disfunction (n=9, 1 SAE); gastrointestinal symptoms (n=1 SAE); infections (n=1)	^#^0.00 Others: ns	
**Liang Q et al. (2017)** (35)	TAC (0.05–0.1 mg/kg/day, target through level 5–10 ng/ml for the first 6 months, then 4–6 ng/ml for 3 months before starting the tapering). Overall duration: 12 months (n=30) IV CYC (0.5–0.75 g/m^2^) monthly for the first 6 months, then every 2-3 months plus oral corticosteroids (1mg/kg/day). Overall duration: 12 months (n= 28)	Median: 10 (range, 0.2–18) after the end of the treatment Median: 10.5 (0.3–19) after the end of the treatment	24/30^$^ 23/28^$^	ns	3/24^%^ 0/23^%^	NA	Glucose intolerance/diabetes mellitus (n=7); leukopenia/anemia (n=2); liver disfunction (n=2); gastrointestinal disturbances (n=2); UTI (n=1)^#^ Glucose intolerance/diabetes mellitus (n=19); UTI (n=8)^#^; leukopenia/anemia (n=8); transaminase elevation (n=6); gastrointestinal disturbances (n=5); pneumonia (n=1)	^#^0.01 Others: ns	
**Ramachandran R et al. (2017)** (30)	TAC (0.1 mg/kg/day, target trough levels 5–10 ng/ml in the first 6 months and 4–8 ng/mL in the next 6 months) for 12 months and oral PDLN (0.5 mg/kg/day) for 6 months, then tapered (n=35) iv MPDN (1 g/day) for 3 days followed by oral PDLN (0.5 mg/kg/day) for 27 days alternate to oral CYC (2 mg/kg/day) for one month. Overall duration: 6 months (n=35)	24 from baseline	21/35* 30/35*	0.03	10/23* 2/31*	0.0069	Infections (n=16); nephrotoxicity (n=8)^#^; diabetes mellitus (n=6); gastrointestinal disturbances (n=5); tremor (n=4); hypertension (n=3) Infections (n=13); amenorrhea (n=5) ^#^; diabetes mellitus (n=5); leukopenia (n=4); gastrointestinal disturbances (n=3); hypertension (n=2)	^#^<0.05 Others: ns	
**Di J et al. (2018)** (36)	PDN (0.5 mg/kg/day for 2 months, then tapered to 10 mg/day) and TAC (0.1 mg/kg/day, target through level 5–10 ng/ml for the first 6 months, then 2 – 4 ng/ml). Overall duration: 12 months (n=38) PDN (0.5 mg/kg/day for 2 months, then tapered to 10 mg/day) and TAC (0.1 mg/kg/day, target through level 5–10 ng/ml for the first 6 months, then 2 – 4 ng/ml). Overall duration: 24 months (n=38)	24 months	25/36* 30/36*	<0.05	8/25^%^ 4/30^%^	<0.05	6 SAEs (infections n=5 pts, of whom 3 died, and interstitial lung disease n=1); glucose intolerance (n=1) Overall incidence: 13.1% vs 15.8%	ns	
**MMF versus supportive and active treatments**									
**Chan TM et al. (2007)** (37)	MMF 1g bid and PDLN (0.8 mg/kg/day tapered until reaching 10 mg/day at around 4 months, then tapered by 2.5 mg/day every 2 weeks until withdrawal). Overall duration: 6 months (n=11) IV MPDN (1g/day) for three days followed by oral PDLN (0.4 mg/kg/day for 3 weeks, then 0.2 mg/kg till the end of the months) alternate to chlorambucil (0.2 mg/kg/day) for one month. Overall duration 6 months. (n=9)	15 from baseline	7/11^%^ 6/9^%^	ns	2/7^%^ 1/6^%^	ns	Dyslipidemia (n=6); infections (n=3); diabetes mellitus (n=1) Leukopenia (n=6)^#^; dyslipidemia (n=5); infections (n=2); nausea and vomiting (n=1); diabetes mellitus (n=1)	^#^0.002 Others: ns	
**Dussol B et al. (2008)** (38)	MMF 2g/day for 12 months (n=19) Supportive treatment (n=17)	12 from baseline	7/19* 7/17*	ns	NA	NA	Myalgias (n=4); gastrointestinal disturbances (n=3); anemia (n=2); infections (n=1); cough (n=1); hypotension (n=1); bullous dermatosis (n=1); neoplasia (n=1) Myalgias (n=5); cough (n=2); gastrointestinal disturbances (n=1); anemia (n=1); infections (n=1); hypotension (n=1)	NA	
**Senthil Nayagam L et al. (2008)** (39)	MMF 1g bid for 6 months and PDLN (0.5 mg/kg/day for 8–12 weeks) (n=11) IV MPDN (1g/day) for three days followed by oral PDLN for 27 days alternate to oral CYC (2mg/kg/day) for one month. Overall duration 6 months. (n= 10)	Mean: 18 (range, 15 – 21) Mean: 16 (range, 13 – 19)	7/11^%^ 8/11^%^	ns	0/7^%^ 1/8^%^	NA	Infections, liver disfunction, gastrointestinal symptoms, cytopenia (number of pts NA)	NA	
**Multitarget therapy versus active treatments**									
**Nikolopoulou A et al. (2019)** (40)	TAC (2 mg bid, target through levels 5–12 ng/ml) combined with MMF (500 mg bid, target blood levels 1.5–3 mg/L); after 1 year of remission, MMF was withdrawn, and TAC was tapered over 6 months. Max duration of treatment: 24 months (n=20) TAC (2 mg bid, target through levels 5–12 ng/ml); withdrawn over 6 months after 1 year of remission. Max duration of treatment: 24 months (n=20)	Median: 71 (range, 9 – 106) Median: 72 (range, 6 – 106)	19/20^%^ 16/20^%^	ns	8/19^%^ 8/16^%^	ns	Gastrointestinal disturbances (n=2); bleeding/anemia (n=2); cholestatis (n=1); haemorrhoidectomy (n=1) Gastrointestinal disturbances (n=3); AKI (n=3); CNI toxicity (n=2); haematuria/UTI (n=2); blackout (n=1); headache (n=1); infections (n=1); gouty arthritis (n=1)	NA	
**Rituximab versus supportive and active treatments**									
**Dahan K et al. (2017)** (41)	RTX 375 mg/m^2^ at time 0 and at 1-week (n=37) Supportive treatment (n=38)	Median: 17 (IQR, 12.5–24.0) Median: 17 (IQR, 13.0–23.0)	24/37^%^ 13/38^%^	<0.01	NA	NA	Cardiovascular disorders (n=4); infections (n=1); edema (n=1); pain/fever (n=1); diarrhea (n=1) AKI (n=2); cardiovascular disorders (n=2); pleural effusion (n=1); malignancy (n=1); edema (n=1); asthma (n=1)	ns	
**Fervenza FC et al. (2019)** (42)	RTX 1g on days 1 and 15 (repeated at month 6 if proteinuria reduced more than 25% without experiencing a complete response) (n=65) CyA (3.5 mg/kg/day, target trough levels 125–175 ng/ml) for 12 months, then tapered and discontinued in 2 months (n=65)	24 from baseline	39/65* 13/65*	<0.001	2/39* 18/34*	NA	179 AEs; the most common were: infections (n=22); infusion reactions (n=22) ^#^; pruritus (n=8) ^#^ 218 AEs; the most common were: gastrointestinal disturbances (n=24)^#^; infections (n=20); worsening of renal function (n=17)^#^	^#^p<0.05 Others: ns	
**Fernandez-Juarez et al. (2021)** (43)	IV MPDN (1 g/day) for 3 days followed by oral PDN (0.5 mg/kg/day) for 27 days alternate to oral CYC (2 mg/kg/day) for one month. Overall duration: 6 months (n=43) TAC (0.05 mg/kg/day, target trough levels 5–7 ng/ml) for 6 months, then tapered by month 9, plus RTX 1g at day 180 (n=43)	24 from baseline	36/43* 25/43*	0.002	1/36^%^ 3/25^%^	NA	239 AEs; the most common were: leukopenia (n=22); infections (n=14); cushing syndrome (n=8) 170 AEs; the most common were: AKI (n=16); infections (n=14); diarrhea (n=13); hyperkalemia (n=6)	0.04	
**Scolari F et al. (2021)** (44)	RTX 1g on days 1 and 15 (n=37) IV MPDN (1g/day) for three days followed by oral PDN (0.5 mg/kg/day) for 27 days, alternate to oral CYC (2 mg/kg/day) for one month. Overall duration 6 months. (n= 37)		17/20* 16/22*	ns	3/23^%^ 6/27^%^	ns	Infusion reaction/drug intolerance (n=10)^#^; infections (n=6); malignancy (n=2); cardiovascular events (n=1); stroke (n=1); glucose intolerance (n=1) Infections (n=15); leukopenia (n=6); glucose intolerance (n=1); cardiovascular events (n=1); nausea/vomiting (n=1); infusion reaction/drug intolerance (n=1); malignancy (n=1)	^#^<0.01 Others: ns	

*, at the end of the follow-up; ^£^, assessed; ^$^, after the first treatment; ^%^, cumulative; ^#^, different in a statistically significant way compared to the control group (for the p value, see within the text); AE, adverse event; AKI, acute kidney injury; bid, bis in die; BSA, body surface area; CR, complete remission; CyA, ciclosporine A; CNI, calcineurin inhibitors; CYC, cyclophosphamide; IV, intravenously; MMF, mycophenolate mofetil; MPDN, methylprednisolone; NA, not available; ns, not significant; PDN, prednisone; PDLN, prednisolone; PR, partial remission; RTX, rituximab; TAC, tacrolimus; SAE, severe adverse event; UTI, urinary tract infection

## Old Therapies

### Corticosteroids or Alkylating Agents Versus Supportive Treatment

Glucocorticoids have been the first immunomodulatory drug employed for the management of PMN. Three randomized controlled trials failed to show significant benefits of prednisone in PMN and, when a benefit was shown, this was not sustained during follow-up (19–21). Moreover, the study demonstrating better response rates in the glucocorticoids arm included a higher proportion of patients with nonselective proteinuria in the placebo arm (21). This has to be interpreted in the context of retrospective, observational studies with controversial results, with some manuscripts reporting good remission rates (45, 46) and others showing that only a small subset of patients obtained transient and not sustained remission (47–49).

Other immunosuppressive drugs were explored in monotherapy for PMN at the early stages of clinical research in the field. A small trial randomized 22 participants with PMN to receive either cyclophosphamide or symptomatic therapy for one year. At the end of this period, no significant differences were detected between the two groups in terms of proteinuria and renal function (18). Alkylating agents were employed also in retrospective studies with contrasting results, with significant heterogeneity across different reports (45, 47–49). Of note, a safety signal emerged from a French cohort of heterogenous glomerular diseases treated with chlorambucil for at least one year: of 41 participants, 3 developed cancer (50). These controversial results supported the idea that a combination of different immunomodulatory drugs may be required for the management of PMN.

### Corticosteroids and Alkylating Agents Versus Supportive Treatment

In 1984, a novel therapeutic approach was proposed. An Italian multicenter, prospective randomized trial assigned 67 patients with PMN and nephrotic syndrome to receive symptomatic treatment or 6 months of a cyclical therapy, with glucocorticoids and chlorambucil administered on alternate months. The month with glucocorticoids consisted of 1 g pulse of IV methylprednisolone repeated for three consecutive days, followed by oral prednisone 0.5 mg/kg for 27 days; then, glucocorticoids were stopped and oral chlorambucil (0.2 mg/kg/day) was given daily for one month. Patients with serum creatinine >1.7 mg/dL were excluded. After a mean follow-up of 30 months, 12/32 (37.5%) treated participants were in complete remission, and another 11/32 (34%) in partial remission, as compared with 9/30 (30%) complete or partial remission in the control group. Importantly, this was associated with a stabilisation of kidney function in the treated group. Among treated participants, one developed obesity and one had reversible increase in serum transaminases (22). The benefits of such an approach were also confirmed in the long term: a study with a median follow-up of 5 years demonstrated that the cyclical therapy led to more frequent and sustained remissions of nephrotic syndrome, in comparison with symptomatic treatment (23) The long-term follow-up of the 1984 study showed that the probability of being still alive and free from ESRD, as well as in complete or partial remission after 10 years, was higher in the cyclical therapy group compared to controls (92% vs 83% and 60% vs 30%, respectively) (15).

The efficacy of the cyclical therapy has been further confirmed in an Indian randomized controlled trial, reporting the 10 years outcomes of 93 patients assigned either to a 6-month regimen alternating glucocorticoids and cyclophosphamide or to symptomatic therapy. Survival without dialysis was 89% versus 65%, respectively. At the last follow-up, 62% of the treated participants had complete (32%) or partial remissions, compared with 35% of the controls. The treated arm experienced fewer infections as well as lower blood pressure and serum cholesterol levels and a higher quality of life. No case of malignancy was reported (24).

### Corticosteroids and Alkylating Agents Versus Active Treatment

In another trial, the effects of the cyclical therapy were compared to those of glucocorticoids alone given with the same schedule and cumulative dosage. After a mean follow-up of 54 months, 28/45 (62%) participants in the cyclical therapy were in complete (30.5%) or partial remission, while, among the 47 participants assigned to glucocorticoids, only 18 (38%) were in complete (17%) or partial remission. Two patients died of cancer, one in the chlorambucil and one in the glucocorticoids group. Other reversible side effects occurred in 9 patients assigned to the cyclical therapy and in 8 assigned to glucocorticoids (25).

Due to concerns related to the potential side-effects of chlorambucil, a randomized controlled trial explored the non-inferiority of cyclophosphamide in the context of the cyclical therapy. In this open-label trial, 87 patients were assigned to receive a 6-month treatment, alternating every other month glucocorticoids with chlorambucil (0.2 mg/kg/24h) or cyclophosphamide (2 mg/kg/24h), respectively. After a median follow-up of 36 months, 12 participants of the 44 assigned to the chlorambucil arm (27%) achieved complete and 24 (55%) partial remission. Among the 43 participants randomized to cyclophosphamide, after a median follow-up of 42 months, 16 achieved complete (37%) and 24 (56%) partial remission. Among responders, 11 patients in the chlorambucil group (30%) and 10 in the cyclophosphamide group (25%) had a relapse, that responded to re-treatment. Six patients in the chlorambucil arm and two in the cyclophosphamide group withdrew treatment due to side-effects; four patients in the chlorambucil group but none in the cyclophosphamide one developed herpes zoster, one patient presented with laryngeal carcinoma four years after chlorambucil therapy, and one developed prostate carcinoma five years after cyclophosphamide therapy. Overall, both treatments showed similar efficacy, but cyclophosphamide appeared to be better tolerated (26). Another prospective randomised study in 32 patients compared two different cyclical regimen treatments: one based on glucocorticoids and chlorambucil, at a lower dose compared to previous studies, and one based on glucocorticoids and cyclophosphamide; the latter was associated with higher response rates and a lower risk of progression towards ESRD (27). Since then, cyclical therapy with cyclophosphamide became more widely employed (51–54).

The role of a cyclophosphamide-based immunosuppressive regimen has also been explored in patients with reduced kidney function. A multi-center randomized controlled trial undertaken in 37 renal units across the UK enrolled 108 patients with deteriorated kidney function, having a creatinine of less than 300 μmol/L (3.4 mg/dl) and at least a 20% decline in renal function measured in the 2 years before study entry. 33 patients received a cyclical therapy with prednisolone and chlorambucil, 37 cyclosporine, and 38 supportive therapy alone. Risk of further 20% decline in kidney function was significantly lower in the prednisolone and chlorambucil group than in the supportive care arm, while this was not significantly different between cyclosporine and supportive treatment; however, serious adverse events were more frequent in the prednisolone and chlorambucil group (29).

In a retrospective study, 9 patients with a baseline serum creatinine ranging from 135 μmol/L to 356 μmol/L (1.5-4 mg/dl) were treated with cyclophosphamide (1 to 2 mg/kg) and 6 of them received concurrent prednisone; they were compared with 17 controls (14 of whom received also prednisone). After a mean follow-up of 83 months, 4 of 9 treated patients achieved a complete remission and 5 a partial one. 1 out of 9 patients in the treatment group (11%) and 10 of the 17 controls (59%) reached ESRD (55). Another group prospectively treated 65 patients with PMN and renal failure (serum creatinine >135 micromol/l, 1.5 mg/dl) with oral cyclophosphamide (1.5-2.0 mg/kg/day for 12 months) and glucocorticoids (methylprednisolone pulses 3 x 1 g, i.v. at months 1, 3 and 5, and oral prednisone 0.5 mg/kg/48 h for 6 months). After a median follow-up of 51 months, 16 patients were in complete and 31 in partial remission, 8 had persistent nephrotic syndrome, one mild proteinuria; of note, 4 patients progressed to ESRD and 5 died. Overall kidney survival was 86% after 5 and 74% after 7 years, compared to 32% after 5 and 7 years in an historical control group. Treatment-related complications occurred in two-thirds of patients, mainly consisting of bone marrow depression and infections. One patient developed bladder cancer and one prostate cancer (56). An alternative regimen has been proposed by Brunkhost et al, who treated 17 PMN patients with a 6-month cyclical therapy scheme, where methylprednisolone was given at a dose of 0.5 g and chlorambucil at a reduced dose of 0.12 mg/kg. After one-year, serum creatinine decreased from 162 to 127µmol/L (1.8 to 1.4 mg/dl) and proteinuria from 16.9 to 5.5 g/d. Side effects were rare and mild (57).

## Therapies in the New Millennium

In 2012 the KDIGO guidelines for PMN recommended that initial therapy should consist of a 6-month course of alternating monthly cycles of oral and intravenous glucocorticoids, and oral alkylating agents. In order to reduce the risk of toxicity, the doses of cyclophosphamide or chlorambucil should be adjusted according to patients’ age and estimated glomerular filtration rate (eGFR) (58).

Despite that, and mainly due to the non-negligible risk of toxicity of a cyclical therapy approach, in the last 20 years several new treatment options have been proposed for PMN.

### ACTH

Intramuscular administration of natural adreno-corticotropin hormone (ACTH) was one of the earliest treatments used for managing idiopathic nephrotic syndrome in children. In 2004, Berg and Arnadottir reported that synthetic ACTH, 0.75–1 mg twice weekly for nine months, allowed to achieve complete remission in 15 patients with MN and nephrotic syndrome, which was sustained for up to 18–30 months in 14 patients (59). A small randomized controlled trial compared the six-month cyclical regimen, based on glucocorticoids alternated to an alkylating agent, with intramuscular synthetic ACTH given at a dose of 1 mg twice a week for one year. In the first group, 15 of 16 participants entered complete or partial remission as a first event, versus 14 of 16 in the second group. Median proteinuria decreased from 5.1 g/day to 2.1 g/day and from 6.0 g/day to 0.3 g/day, respectively, in the two arms. No significant side effects were seen in participants assigned to ACTH (28). However, it has to be noted that, although mitigated, ACTH side effects are potentially the same as glucocorticoids.

The role of ACTH in PMN has been further explored in other studies. In a retrospective series, 17 patients were treated with synthetic ACTH for nine months, four patients entered complete remission and seven partial remission. These results were inferior to those observed in historical controls treated with oral cyclophosphamide for one year (60). In the United States, the effects of a natural ACTH gel formulation were assessed in 11 patients with PMN. Two participants entered complete and seven a partial remission, while two failed to respond (61). In another study, 20 patients with MN and nephrotic syndrome received a subcutaneous dose of 40 or 80 IU ACTH twice weekly. At 12 months, proteinuria decreased from 9.1 g/day to 3.9 g/day, with improvement in serum albumin and cholesterol. No significant adverse effects were documented (62). Despite these encouraging results, evidence for a role of ACTH in PMN is still relatively weak and more data are needed. Also, the mechanism of action of ACTH in this context is unclear. It has been hypothesized that this may depend upon activation of melanocortin receptor-1 (MCR-1), which is co-localized with synaptopodin in podocytes. MCR-1 might interfere with catalase and RHO-1 protein activity, consequently regulating cytoskeletal stability and preventing podocyte apoptosis (63). However, it has been shown that, in MCR1-null mice, melanostimulating hormone can reduce proteinuria and protect podocytes from lipopolysaccharide injury *via* a MCR1-independent mechanism (64). Moreover, it is possible that the effects of ACTH are modulated by β-defensins, a new class of melanocortin ligands that can cross talk between MCRs and the immune system (65).

### Calcineurin Inhibitors (CNIs)

In the early 1980s, the discovery of cyclosporine revolutionized the treatment of allotransplantation. A few years later, the role of this drug was also explored in PMN. Several observational studies reported a decline in proteinuria from nephrotic to non-nephrotic range and even complete remission in patients with PMN (66–69). A review of 73 patients with PMN who received cyclosporine reported complete remission in 20% of cases, partial remission in 25% and failure in 55% (70). However, disease flares were frequent when cyclosporine was withdrawn or reduced. Moreover, the potential nephrotoxicity of cyclosporine, which is dose- and time-dependent, can raise concerns regarding this treatment option. Two prospective randomised controlled studies investigated the role of cyclosporine in PMN. In a study published in 1995, 17 patients with PMN and worsening of kidney function, after a run-in phase of 1 year, were randomised to cyclosporine or placebo. Treatment with cyclosporine was associated with a significant reduction in the eGFR decline and a sustained improvement in proteinuria. Of note, only one patient per arm received renin-angiotensin-system (RAS) inhibitors (31). A second study compared a 26 week course of cyclosporine and low-dose prednisone (28 patients) with placebo and low-dose prednisone (23 patients). At 26 weeks (primary end-point), remission occurred in 75% versus 22% (p<0.001). However, during or after the tapering, 48% and 60% of the patients in remission in the cyclosporine and placebo arms, respectively, relapsed. Remarkably, only 19 patients received RAS-inhibitors during the study (1 cyclosporine and 8 placebo) (33).

Around 10 years after cyclosporine discovery, tacrolimus, another calcineurin inhibitor, was approved for prevention of rejection in organ transplantation. Like cyclosporine, the role of tacrolimus has also been examined in PMN. A few observational studies reported a good rate of partial remissions; however, as with cyclosporine, relapses were frequent after drug withdrawal (71).

Two big retrospective studies supported a role for tacrolimus in the management of PMN. A Spanish multicenter group reported the outcomes of tacrolimus monotherapy at a mean dose of 0.05 mg/kg/d in 122 PMN patients with nephrotic syndrome and stable kidney function. After a mean treatment duration of 17.6 ± 7.2 months, including a full-dose and a tapering period, 102 (84%) patients responded. Among responders, 42% achieved a complete and 58% partial remission (72). Another large Chinese study described outcomes in 408 consecutive patients with PMN and nephrotic syndrome treated with tacrolimus. The cumulative partial or complete remissions after therapy were 50% at 6 and 67% at 24 months. The cumulative complete remission rates were 4%, and 23%, respectively. A relapse occurred in 101 of the 271 (37.3%) patients (73).

The role of tacrolimus in PMN has been further established in randomized controlled trials, that compared it with either supportive or other active treatment. A Spanish controlled trial reported a high remission rate in patients assigned to tacrolimus, in comparison with untreated controls after 18 months of therapy (94% versus 35%), although 50% of the patients relapsed after tacrolimus withdrawal (32). In a Chinese multicenter trial, 73 patients with nephrotic PMN were randomized to tacrolimus plus prednisone for 9 months or cyclophosphamide plus prednisone for 4 months. Remission was reached earlier with tacrolimus, but at 12 months the remission rate was comparable in the two groups. Of note, patients receiving tacrolimus were more likely to develop diabetes, infection, and hypertension (34).

Another Chinese study explored the efficacy of a 12 month course of tacrolimus, compared with a 12 month course of cyclophosphamide and glucocorticoids. During the first year of follow-up, the probability of remission and the average time to remission were similar between the two groups. Relapses occurred only in the tacrolimus group in 3 patients after drug withdrawal, and 2 out of 3 were successfully retreated using the same scheme. The through levels of tacrolimus were significantly lower in non-responders, compared with patients reaching complete or partial response (3.1 ± 1.1 ng/ml versus 5.8 ± 1.6 ng/ml and 4.8 ± 2.1 ng/ml; p <0.05). The safety profile was better in the tacrolimus group, especially in terms of infections (35).

A single-center randomized trial compared the effects of a 12 month course of tacrolimus plus prednisone versus a cyclical therapy with cyclophosphamide alternated with glucocorticoids in 70 patients with PMN and persistent nephrotic syndrome. At 12 months, remission rates were comparable (71% with tacrolimus vs 77% with cyclical therapy), while at 24 months, 43% of the patients assigned to tacrolimus and 80% of patients assigned to cyclical therapy were in remission. Patients on cyclophosphamide had a significantly higher risk of amenorrhea while those on tacrolimus experienced a greater risk of reversible nephrotoxicity (30).

A Chinese group compared a 12 and a 24 month course of tacrolimus plus glucocorticoids in 76 patients. At the 24 month assessment, the longer course was associated with higher remission rates and a lower incidence of relapses (p <0.05). Of note, six patients did not complete the treatment protocol because of pulmonary infections, that were fatal in 3 of them (36).

In summary, CNIs may be a useful therapeutic option for nephrotic patients with well-preserved kidney function. Most patients experience a remission with a significant reduction in the risk of deteriorating kidney function, however the relapse risk is high when this class of drugs is discontinued.

### Mycophenolate Salts

A role for mycophenolate salts has also been proposed in MN. Observational studies involving small numbers of patients reported that mycophenolate mofetil (MMF), usually associated with prednisone, reduced proteinuria. In a retrospective study, MMF, 1 g twice daily, for 12 months was compared to cyclophosphamide, 1.5 mg/kg/d for 12 months. Both groups also received intermittent methylprednisolone pulses and alternate-day prednisone. Cumulative incidence of remission (66% vs 72%) and side effects (75% vs 69%) were similar, but the relapse risk was greater with MMF compared to cyclophosphamide (38% vs 13%) (74). A prospective, controlled, open-label study randomized 20 patients with MN to receive either the association of MMF and prednisolone for 6 months or a cyclical regimen. Remission (complete or partial) rates were 63.6% in the MMF group and 66.7% in the cyclical treatment group, and serum creatinine remained stable during a mean follow up of 15 months. Nephrotic proteinuria relapsed in two patients assigned to MMF and in one to cyclical therapy; chlorambucil resulted in higher risk of leukopenia compared to MMF (37). Similarly, another trial compared the efficacy of MMF with cyclical therapy in 21 nephrotic adults with MN. Of the 11 participants randomized to receive MMF (2 g/day for 6 months) and oral prednisolone (0.5 mg/kg/day for 2-3 months), 7 (64%) achieved complete or partial remission, compared to 8/10 (80%) treated with a 6-month regimen of glucocorticoids alternated with cyclophosphamide every other month (39).

In another controlled trial, 36 patients with primary MN were randomized to MMF (2 grams per day) for one year or symptomatic therapy. At 12 months, there was no difference between the two groups in terms of mean proteinuria reduction, as well as in terms of rate of complete and partial remissions. Serious adverse effects were observed in 4 of 19 (20%) patients receiving MMF (38). A more recent trial assigned 40 patients with PMN and nephrotic syndrome to receive either tacrolimus monotherapy or tacrolimus combined with MMF for 12 months. At the end of the follow-up, 16/20 (80%) patients in the tacrolimus group achieved remission compared to 19/20 (95%) in the tacrolimus/MMF group. Of note, no difference was detected in terms of relapse rate between groups (50% vs 42%, respectively) (40).

In summary, a role for MMF in improving proteinuria has been documented, at least in the short term and in the context of small studies. However, complete remissions are rare, relapses are frequent and long-term benefits are yet to be clarified.

### Rituximab – Uncontrolled Experience

Rituximab is a chimeric human/murine monoclonal antibody that binds CD20, a membrane protein expressed on B cells, and induces killing of CD20+ B-cells. The efficacy of rituximab in PMN was initially tested in 9 patients treated with the dose of 375 mg/m^2^ every week for 4 weeks: proteinuria decreased from a mean of 8.6± 1.4 g/day to 3.8± 0.8 g/day after 4 weeks (75). In a multi-center study, rituximab at a dose of 375 mg/m^2^ every week for 4 weeks was administered to 20 patients with MN and proteinuria > 5 g/day, and treatment was repeated after 6 months (76). Two patients did not respond to the first course and 18 completed the treatment, with proteinuria decreasing from 11.9 to 2.0 grams per day. At the last visit, 4 patients were in complete remission (20%), 12 in partial (60%), 1 had limited response and 1 relapsed. Beck et al. administered rituximab to 25 patients with anti-PLA2R antibodies positive MN; in 17 patients the antibodies declined or disappeared within 12 months after rituximab, obtaining the so called “immunological response”. Five of them (29%) achieved complete and 10 (59%) partial remission at 2 years. Among the 8 patients with persistently elevated levels of anti-PLA2R antibodies, none achieved remission after 1 year and 3 experienced partial remission at 2 years (77). A multi-center study collected data of 23 patients with PMN treated with rituximab. At 12 months, complete remission was achieved in 6 (26%) patients and partial in 13 (overall renal response, 82.6%). In 3 patients, nephrotic syndrome relapsed 27-50 months after treatment. Importantly, eGFR <45/ml/min/1.73 m^2^ was an independent risk factor for rituximab failure (78). The largest uncontrolled series of PMN patients treated with rituximab was collected by the Bergamo group. Out of 132 patients with MN treated with rituximab and followed for a mean time of 30 months, 84 responded (63.6%), with 43 (32.6%) achieving complete and 41 (31.0%) partial remission. Among the responders, 25 (30%) had a relapse of nephrotic syndrome, with a higher risk of disease flares in patients who achieved partial remission, compared to the ones that achieved complete remission (50% versus 30%). The response rate was similar regardless of anti-PLA2R antibodies positivity; however, re-emergence of circulating antibodies predicted relapse of the disease (79). No treatment-related serious adverse events were reported, although, in a previously published cohort of 100 PMN patients treated with rituximab, the same group described four deaths, three patients who developed cancer, four progressions to ESRD and 8 patients with serious cardiovascular events. However, it has to be noted that these adverse events were observed in the context of a significant burden of previous immunosuppression (80).

The use of rituximab in PMN has also been studied in combination with other immunomodulatory approaches. A cohort of 10 patients with MN and proteinuria > 10 grams per day was treated with rituximab, plasmapheresis and iv immunoglobulins, with achievement of partial remission in 90% of the cases (81). In another observational study, 15 patients with MN received a combination treatment with oral cyclophosphamide for 8 weeks, prednisone at 60 mg daily, slowly tapered at 6 months, and rituximab 1000 mg 2 weeks apart followed by 1000 mg every 4 months for two years. Among treated patients, 93% achieved complete remission at a median time of 13 months. Three patients experienced reversible serious side effects: severe neutropenia and viral infection in two cases and altered mental status in one patient (82). A meta-analysis of 8 studies including 542 patients with MN showed that, in comparison with controls who received different treatments, rituximab improved the total remission rate, achieving a higher rate of complete remission while reducing the anti-PLA2R antibody titre. Adverse events were mostly mild in nature, and serious adverse events rare (83). Of note, an increased risk of severe infections in rituximab treated patients has been found in those with CKD, diabetes, or hypogammaglobulinemia (84–87). Moreover, several studies reported late-onset neutropenia, occurring usually several months following the administration of rituximab (88–90).

The effect of the cumulative rituximab dose on response in PMN is unclear, with conflicting results across different cohorts (91, 92). In a study, different protocols of rituximab were used in patients with PMN. Among 55 participants, 28 received two infusions of rituximab 1g at 2-week intervals, whereas the other 27 participants received two infusions of 375 mg/m^2^ at 1-week interval. Remission occurred in 24 patients (86%) in the first group versus 18 (67%) in the second group and the median time to remission was 3 and 9 months, respectively. This data suggested that higher rituximab dose may be more effective in PMN (93). Another study compared efficacy and safety of 3 different treatment regimens: low-dose rituximab (one dose of 375 mg/m^2^), standard dose (four weekly doses of 375 mg/m^2^) and controls treated with the cyclical regimen. At 24 months, a significant improvement in proteinuria was observed in all groups (from 7.5 g/d to 0.21 g/d in the low-dose rituximab, from 5.1 to 0.35 g/d in the standard-dose rituximab and from 8.27 to 2.2 g/d in the cyclical regimen group) (94). In a recent retrospective study, 60 patients with PMN were treated with rituximab, administered over a 2-year period, combined with an initial short course of low dose oral cyclophosphamide and a rapidly tapered course of prednisone. By 2 years, all patients reached partial remission, and 83% complete remission; response to treatment was durable with 90% of patients remaining relapse-free. In addition, all patients achieved immunological remission by 6 months after starting therapy. Adverse events were infrequent with the most common being late-onset neutropenia (95).

### Rituximab Versus Supportive and Active Treatments

The randomized controlled trial GEMRITUX, including 75 patients with biopsy-proven MN, compared non-immunosuppressive antiproteinuric treatment alone with non-immunosuppressive antiproteinuric treatment plus rituximab 375 mg/m^2^ on days 1 and 8. There was no difference in remission rates at 6 months. However, during the observational phase, complete or partial remission was achieved in 24 of 37 (64.9%) patients in rituximab group versus 13 of 38 (34.2%) controls, with a median time to remission of 7 months. Of note, the “immunological response” of anti-PLA2R antibodies predicted response to treatment. Eight serious adverse events occurred in each group (41). In the randomized controlled trial MENTOR, 130 patients with PMN, proteinuria ≥ 5 g/d and creatinine clearance ≥ 40 ml/min/1.73 m^2^, were randomized to receive intravenous rituximab (two infusions, 1000 mg each, administered 14 days apart; repeated at 6 months in case of signs of partial response) or oral cyclosporine (starting at a dose of 3.5 mg/kg/d for 12 months), after a run-in phase with RAS inhibitors for at least 3 months. Cyclosporine was then tapered over two months (months 12-14) and patients were followed for 24 months. At 12 months, 39 of 65 patients (60%) in the rituximab group and 34 of 65 (52%) in the cyclosporine group had a complete or partial remission, while at 24 months, 39 patients (60%) in the rituximab group and 13 (20%) in the cyclosporine group had a complete or partial remission (p<0.001). Remarkably, the “immunological response” was quicker, more frequent, and more sustained in the rituximab group. Serious adverse events occurred in 11 patients (17%) in the rituximab group and in 20 (31%) in the cyclosporine group (42). The efficacy of rituximab and cyclosporine was therefore similar. However, when cyclosporine was withdrawn, the relapse rate was high, suggesting that the beneficial effect of rituximab is longer in the context of cyclosporine withdrawal (96). This finding, similar to what observed when comparing cyclical therapy and CNIs, may question the role of the latter as first-line agents, at least when the goal of treatment is sustained remission (97).

In another recently published randomized, open-label controlled trial (STARMEN), conducted in Spain and the Netherlands, 86 patients with PMN and persistent nephrotic syndrome after a 6-month observation period were assigned to receive a 6-month cyclical treatment with glucocorticoid and cyclophosphamide or sequential treatment with tacrolimus (full-dose for 6 months and tapering for another 3 months) and a single infusion of rituximab (1 gram at month 6). Primary outcome was complete or partial remission of nephrotic syndrome (composite endpoint) at 24 months (43). The primary outcome occurred in 36 patients (83.7%) in the glucocorticoid-cyclophosphamide group and in 25 patients (58.1%) in the tacrolimus-rituximab group (relative risk [RR] 1.44; 95% confidence interval [CI] 1.08 to 1.92). Complete remission at 24 months occurred in 26 patients (60%) in the glucocorticoid-cyclophosphamide group and in 11 patients (26%) in the tacrolimus-rituximab group (RR 2.36; 95%CI 1.34 to 4.16). The “immunological response” was quicker in the cyclical regimen group and associated with remission at 24 months. Relapses occurred in one patient (2.7%) in the cyclical regimen group, and three (12%) in the tacrolimus-rituximab group. There were more adverse events in the cyclical regimen group, although the rate of serious adverse events was similar in both arms. This study provided evidence that cyclical treatment with glucocorticoid and cyclophosphamide is superior to a combination of tacrolimus and rituximab in inducing persistent remission in PMN. It should be noted that almost 60% of patients treated with tacrolimus-rituximab had a good clinical response, and few responders relapsed after discontinuing tacrolimus. This supports a potential role for rituximab in preventing the occurrence of relapses if administered at the time of calcineurin inhibitors withdrawal (98). The pilot trial RICYLO performed a head-to-head comparison of rituximab (1 g two weeks apart) and cyclical regimen in 74 patients, with the primary outcome of complete remission at 12 months. The study failed to show statistically significant differences in remission rates for the two groups (complete remission 32% vs 16% in the cyclical regimen and rituximab arms, respectively [OR 0.40, 95%CI 0.13-1.23]; combined complete and partial remission 62% and 73% [OR 0.61, 95%CI 0.23-1.63]). The time to complete or complete and partial remission up to month 24 in the two groups was similar (p=0.78 and p=0.47, respectively). No differences in adverse events were detected in the two arms. Taken together, the results of this pilot study suggest a similar effectiveness of the two regimens (44).

## How to Treat Patients With PMN?

Retrospective studies and the recently published randomized control trials showed that the efficacy of rituximab in PMN is similar to the cyclical regimen and CNIs, which have been historically considered as first-line approach in the management of the disease. Moreover, compared to CNIs, rituximab (similarly to the cyclical regimen) is more effective in inducing sustained remission, while the risk of relapse is high when CNIs are withdrawn. In this context, an approach of “consolidation” of remission with rituximab administration at the moment of CNIs tapering may be an attractive option to prevent relapses. Of note, the favourable safety profile of rituximab makes this drug a good first-line candidate for management of the disease. The recently published KDIGO 2021 Guideline for the management of glomerular diseases (99) suggests rituximab or CNIs as first-line approaches for patients at moderate risk of progressive loss of renal function (defined as normal and stable eGFR after diagnosis), while rituximab or cyclical regimen or CNIs with rituximab are recommended for patients at high risk (reduced eGFR, or proteinuria >8g/day, or normal eGFR associated with serum albumin <25 g/l, or PLA2R antibodies >50 RU/ml). On the other hand, the cyclical regimen is advised as first-line approach for patients at very high risk (life-threatening nephrotic syndrome or rapid deterioration of kidney function). The use of a risk stratification approach to guide therapy is indeed a new concept compared to the 2012 KDIGO Clinical Practice Guideline for Glomerulonephritis, where the initial recommended treatment was, for all patients eligible for immunosuppression, a 6 month course of cyclical therapy, with CNIs being the only alternative first-line approach.

Rituximab is therefore due to be considered the first therapeutic line for the majority of patients with PMN, even in the context of reduced, although stable, renal function (100). However, further issues remain still unresolved.

First, it has to be clarified if biomarkers may contribute to inform the treatment strategy. In fact, reports support a higher efficacy of cyclophosphamide-based regimens in inducing “immunological remission”, compared to rituximab, in patients with high anti-PLA2R antibodies titres (101). The best approach in this context is yet to be confirmed; of note inadequate rituximab dose might have played a role.

It is also unclear what the optimal dosing strategy of rituximab is. Importantly, patients with higher proteinuria and lower serum albumin at time of rituximab infusion show lower residual serum rituximab levels at month-3 (102), and this was associated with subsequent lower response rates (103). These findings support the idea that rituximab dose can affect the response, especially in patients with higher proteinuria. However, as already discussed, findings from retrospective studies are contradictory in this respect. The three prospective studies available so far employed different therapeutic strategies (**Table 2**); however, despite all three trials included patients with full-blown nephrotic syndrome, differences in terms of baseline characteristics need to be acknowledged, and prevent the possibility of performing a direct comparison across them. The issue of the optimal dosing regimen therefore remains unresolved. Dedicated prospective studies will be needed, ideally including homogenous cohorts of patients in terms of disease severity, proteinuria, kidney function, anti-PLA2R antibodies titre and histological characteristics.

**Table 2:** Main baseline characteristics, therapeutic schedule and response rates in the rituximab arms of randomized control studies exploring rituximab effectiveness in primary membranous nephropathy.

Study	Reference	Proteinuria	PLA2R-Ab	RTX dose	CR+PR 6 months	CR+PR 12 months
GEMRITUX	Dahan K et al. (2017) (41)	7.7 g/g (4.6-10.4)*	41 (0-276)^$^	375 mg/mq^2^ day 1 and day 8	35.1%	NA^£^
MENTOR	Fervenza FC et al. (2019) (42)	8.4 g/day (6.8-12.3)^$^	409 (163-834)^&^	1 g day 1 and 15; repeated at month 6 if no CR and proteinuria reduction >25%°	35%	60%
RICYCLO	Scolari F et al. (2021) (44)	6 g/day (4-10)^$^	63 (52-87)	1 g day 1 and 15	51%	62%

*: protein to creatinine ratio; median (IQR).

$: Median (IQR).

£: 65% after a median of 17 months (IQR 12.5-24).

&: different ELISA method compared to GEMRITUX and RICYCLO.

°: 37/65 patients retreated.

PLA2R-Ab, phospholipase A2 receptor antibodies; RTX, rituximab; CR, complete remission; PR, partial remission.

Other uncertainties regarding the use of rituximab in PMN are how to identify patients that may experience relapses, and how this subgroup should be managed. For this purpose, the re-appearance of anti-PLA2R antibodies is well defined as predictor of flares (79), although the role of other biomarkers, such as the kinetics of CD20+ B-cells and their subsets, will need to be studied further.

With humanized anti-CD20 monoclonal antibodies becoming more easily available on the market, a rising issue is how to manage rituximab-resistant patients. For rituximab-treated patients with resistant or early relapsing disease, the presence of anti-rituximab antibodies should be considered. In this context, testing of such antibodies could identify patients that may benefit from further rituximab administrations (anti-rituximab antibodies undetectable) (104) or from humanized anti-CD20 monoclonal antibodies (105, 106).

Finally, it is also uncertain how to manage patients with refractory or multi-relapsing disease courses. In this setting, the role of pre-emptive rituximab administration, as well as of alternative immunomodulating approaches with less clear evidence, such as ACTH or MMF, needs to be further investigated.

## Conclusions

PMN is a rare disease that requires immunosuppressive treatment in patients not achieving spontaneous remission. Achievement of remission (ideally complete) is advised in order to reduce the risk of ESRD and the complications of nephrotic syndrome *per se*. Rituximab, CNIs or the cyclical regimen are considered as first-line therapeutic approaches. In keeping also with the recently published KDIGO 2021 Guideline for the management of glomerular diseases (99), the cyclical regimen is to be reserved for patients at high risk of progressive loss of renal function or life-threatening nephrotic syndrome. For patients requiring CNIs, prolonged treatment at low doses needs to be considered, due to the high relapse rate following their withdrawal; the long-term risk of nephrotoxicity of such an approach has to be taken into account. Of note, in the case of CNI withdrawal, administration of rituximab at the time of CNI tapering may reduce the risk of relapse. Further studies are required to confirm the role of other immunomodulatory approaches and to identify patients more likely to benefit from the different first-line therapeutic options available, as well as to determine the ideal rituximab induction regimens and the timing of re-treatment.

## Author Contributions

FS, FA, FM, EDB, HT, MP and CP drafted and reviewed the manuscript. All authors contributed to the article and approved the submitted version.

## Conflict of Interest

FA declares consultancy fees from Baxter, AstraZeneca and Otsuka, advisory board for Trevere Therapeutics. These CIs are not relevant for the topic of the submitted paper.

The remaining authors declare that the research was conducted in the absence of any commercial or financial relationships that could be construed as a potential conflict of interest.

## Publisher’s Note

All claims expressed in this article are solely those of the authors and do not necessarily represent those of their affiliated organizations, or those of the publisher, the editors and the reviewers. Any product that may be evaluated in this article, or claim that may be made by its manufacturer, is not guaranteed or endorsed by the publisher.

## Appendix

Although the definitions of complete and partial remission have been established by international guidelines (58), the different randomized clinical trials in primary membranous nephropathy have applied slight modifications. For instance, the MENTOR trial (42) defined complete remission as proteinuria of no more than 0.3 g/24h and a serum albumin level of at least 3.5 g/dl, whilst the study by Ramachandran et al. (30, 107) used a proteinuria <500 mg/24h with normal serum albumin (≥3.5 g/dl) and serum creatinine. Likewise, the STARMEN (43) employed a proteinuria ≤0.3 g/24h but also included a stable kidney function defined as an estimated glomerular filtration rate (eGFR) ≥45 ml/min/1.73m2 whereas the RI-CYCLO (44) trial only applied the proteinuria parameter (≤0.3 g/24h). On the other hand, partial remission in the MENTOR (42) study was defined as a reduction in proteinuria of at least 50% from baseline plus final proteinuria between 0.3 g–3.5 g/24h regardless of creatinine clearance or serum albumin level, while Ramachandran et al. (30, 107) used a 24h urine protein ≥500 mg/24h, but <2 g/24h or <50% of baseline with normal serum albumin (≥3.5 g/dl) and serum creatinine. Additionally, the STARMEN (43) defined partial remission as a reduction of proteinuria >50% from baseline and a value <3.5 g/24h plus stable kidney function (eGFR ≥45 ml/min/1.73m2) and the RI-CYCLO (44) employed a proteinuria of at least 50% lower than the baseline and ≤3.5 g/24h without including albumin or creatinine levels.
